# Position statement on infection screening, prophylaxis, and vaccination in pediatric patients with rheumatic diseases and immunosuppressive therapies, part 2: infection prophylaxis

**DOI:** 10.1007/s00431-023-05080-3

**Published:** 2023-07-13

**Authors:** Daniel Clemente Garulo, Esmeralda Núñez-Cuadros, Marisol Camacho Lovillo, Joan Calzada-Hernández, Sara Guillén Martín, Laura Fernández Silveira, María José Lirola Cruz, Alfredo Tagarro, Rosa María Alcobendas Rueda, Agustín López López, Miren Satrustegi Aritziturri, Cristina Calvo

**Affiliations:** 1 Pediatric Rheumatology Unit, Hospital Infantil Universitario Niño Jesús, Madrid, Spain; 2 Pediatric Rheumatology Unit, UGC Pediatría, Hospital Regional Universitario de Málaga, Instituto de Investigación Biomédica de Málaga (IBIMA), Av. del Arroyo de los Ángeles, S/N, 29011 Málaga, Spain; 3 Servicio de Inmunología, Hospital Universitario Virgen del Rocío/Instituto de Biomedicina de Sevilla (IBIS), Reumatología E Infectología Pediátricas, Seville, Spain; 4 Unitat de Reumatologia Pediàtrica, Servei de Pediatria, Hospital Sant Joan de Déu, Esplugues de Llobregat, Barcelona, Spain; 5 Department of Paediatrics, Hospital Universitario de Getafe, Getafe, Madrid, Spain; 6 Department of Paediatric Rheumatology, Instituto Hispalense de Pediatría, Seville, Spain; 7 Pediatrics Department, Instituto de Investigación 12 de Octubre (imas12), Hospital Universitario Infanta Sofía, Universidad Europea, Hospital 12 de Octubre, Madrid, Spain; 8 Department of Pediatric Rheumatology, La Paz Children’s Hospital, Madrid, Spain; 9 Department of Paediatrics, Hospital Universitario Puerta de Hierro, Majadahonda, Madrid, Spain; 10 Servicio de Pediatría, Hospital Universitario Donostia, San Sebastián, Spain; 11 Department of Pediatrics, Infectious and Tropical Diseases, Hospital Universitario La Paz, and La Paz Research Institute (IdiPaz), Madrid, Spain; 12 Translational Research Network of Pediatric Infectious Diseases (RITIP). Madrid, Madrid, Spain

**Keywords:** Vaccination, Prophylaxis, Tuberculosis, Varicella, Herpes zoster virus, *Pneumocystis jirovecii*, Immune-mediated rheumatic diseases, Consensus

## Abstract

This study aims to provide practical recommendations on prophylaxis for infection in pediatric patients with immune-mediated rheumatic diseases receiving/scheduled to receive immunosuppressive therapy. A qualitative approach was applied. A narrative literature review was performed via Medline. Primary searches were conducted using MeSH terms and free text to identify articles that analyzed data on infections and vaccinations in pediatric patients with immune-mediated rheumatic diseases receiving immunosuppressive therapy. The results were presented and discussed in a nominal group meeting comprising a committee of 12 pediatric rheumatologists from the Prevention and Treatment of Infections Working Group of the Spanish Society of Pediatric Rheumatology. Several recommendations were generated. A consensus procedure was implemented via a Delphi process that was extended to members of the Spanish Society of Pediatric Rheumatology and the Vaccine Advisory Committee of the Spanish Association of Pediatrics. Participants produced a score ranging from 0 (completely disagree) to 10 (completely agree). Agreement was considered to have been reached if at least 70% of participants voted ≥ 7. The literature review included more than 400 articles. Overall, 63 recommendations were generated (23 on infection prophylaxis) and voted by 59 pediatric rheumatologists and other pediatric specialists, all of whom achieved the pre-established level of agreement. The recommendations on prophylaxis of infection cover vaccination and prophylaxis against varicella zoster virus, tuberculosis, *Pneumocystis jiroveccii*, and invasive fungal infections in pediatric patients with immune-mediated rheumatic diseases receiving/scheduled to receive immunosuppressive therapy.

*Conclusion*: Based on current evidence and a Delphi process, we provided consensus and updated recommendations on prophylaxis and treatment of infections to guide those caring for pediatric rheumatology patients.

**Table:** 

**What is Known:**
•*Data largely derived from adults find that infectious diseases and related complications are a major cause of morbidity and mortality in patients with immune-mediated rheumatic diseases.*
•*It is crucial to be aware of the preventive measures that should be implemented to prevent these infections in children, although most guidelines are often extrapolated from adult cases.*
**What is New:**
•*In the absence of evidence, a literature review and a Delphi survey were conducted to establish a series of expert recommendations that could prove useful in clinical practice, providing a practical and simple day-to-day approach to be used by pediatric rheumatologists.*
•*The recommendations focus on tuberculosis, herpes zoster virus, fungal infections, and Pneumocystis jirovecii.*

## Introduction

Infectious diseases and related complications are a major cause of morbidity and mortality in adult patients with immune-mediated rheumatic diseases. The increased risk of infection in this population may be related to several factors, including the immune effects of the disease itself, use of immunosuppressive drugs, comorbidities, medical/surgical procedures, as well as frequent visits to the clinic [1].

Like adults, pediatric patients with immune-mediated rheumatic diseases could be at a higher risk of infection than healthy children because of their underlying condition [2]. With current intensive treatment strategies incorporating the early use of immunosuppressive therapies such as biological drugs, high-dose glucocorticoids, and Janus kinase inhibitors, susceptibility to infections, including opportunistic infections, increases [3]. In addition, the fact that children receive many vaccinations during the first years of life could reduce the immunogenicity of vaccinations because of the patients’ immunosuppressed status, thereby further increasing the risk of infection [2]. Consequently, prophylaxis for infection in patients with immune-mediated rheumatic diseases, especially with immunosuppressive treatment, is essential.

The lack of published trials in the immunocompromised pediatric population means that pediatricians do not have comprehensive, up-to-date guidelines. Therefore, we designed this project to generate practical recommendations on screening, prophylaxis, and vaccination against infection in pediatric patients with immune-mediated rheumatic diseases receiving/scheduled to receive immunosuppressive therapy. The recommendations on screening were recently published (4). This article describes the current evidence and relevant recommendations generated on prophylaxis of infection (vaccination, herpes zoster virus, tuberculosis, fungal infections, and *Pneumocystis jirovecii*) in this population. We are confident this guide will help physicians resolve questions that may arise in day-to-day practice, thereby improving pediatric care and outcomes.

## Methods

This qualitative study was based on a comprehensive narrative literature review, the experience of an expert committee, and the consensus achieved by pediatric rheumatologists. The project was carried out following the ethical principles of the Declaration of Helsinki for medical research involving human subjects and in accordance with the stipulations of Good Clinical Practice.

A narrative literature review was performed via Medline. Primary searches were conducted using MeSH terms and free text to identify articles that analyzed data on infections and vaccinations in pediatric patients with immune-mediated rheumatic diseases receiving immunosuppressive therapy. The results were presented and discussed in a nominal group meeting comprising a committee of 12 pediatric rheumatologists from the Prevention and Treatment of Infections Working Group of the Spanish Society of Pediatric Rheumatology. Several recommendations were generated. A consensus procedure was implemented via a Delphi process that was extended to members of the Spanish Society of Pediatric Rheumatology and the Vaccine Advisory Committee of the Spanish Association of Pediatrics. Participants produced a score ranging from 0 (completely disagree) to 10 (completely agree). Agreement was considered to have been reached if at least 70% of participants voted ≥ 7. With the assistance of a methodologist, each recommendation was assigned a level of evidence (LE) and grade of recommendation (GR) according to the recommendations of the Oxford Center for Evidence-Based Medicine [5].

A more detailed description of the process can be found [4]. In this article, the results refer to 23 recommendations that cover vaccination and prophylaxis against varicella zoster virus, tuberculosis, *Pneumocystis jiroveccii*, and invasive fungal infections in pediatric patients with immune-mediated rheumatic diseases receiving/scheduled to receive immunosuppressive therapy.

## Results

### Vaccination

The recommendations generated in this consensus document, as well as the Delphi process results, are depicted in Table 1. A total of 59 experts participated in the Delphi process (response rate, 64%), 45 from SERPE and 14 from SEIP.

**Table 1:** Delphi results*

**#**	**Recommendation**	**Mean**	**SD**	**Median**	**p25**	**p75**	**Min**	**Max**	**LE**	**GR**	**70% ≥ 7**
**1**	Triple viral vaccination (measles, mumps, rubella (MMR)) is recommended, whenever possible, at least 2–4 weeks before starting immunosuppressive treatment if not previously vaccinated.	9.46	3.54	10	9	10	5	10	IIIb	D	98%
**2**	If serology testing for varicella zoster is negative, varicella vaccination is recommended, whenever possible, 2–4 weeks before starting immunosuppressive treatment.	9.54	0.79	10	9	10	7	10	IIIb	D	100%
**3**	Children 6 months or older with rheumatic disease should be vaccinated against influenza during the flu season, especially if they are receiving immunosuppressive treatment. Vaccination of the entire family is recommended.	9.71	1.08	10	10	10	3	10	IIIb	D	98%
**4**	Prophylactic treatment for chickenpox is recommended after contact with a person with varicella zoster infection in children with rheumatic dise.ases receiving medium- and high-risk immunosuppressive treatments who do not have a previous history of chickenpox or herpes zoster, vaccination, or evidence of immunity confirmed by serology testing	8.76	1.41	9	8	10	3	10	IIIb	D	91%
**5**	Prophylaxis with varicella zoster immunoglobulin or intravenous non-specific immunoglobulin within 10 days (preferably within 7 days) after exposure is recommended in patients considered at high risk of severe disease.	8.93	1.42	9	8	10	4	10	IIIb	D	93%
**6**	In the event of close contact with a person with active tuberculosis, the tuberculin skin test (TST) and interferon gamma release assay (IGRA) should be performed, as should a chest X-ray.	9.61	0.70	10	9	10	7	10	IIIB	D	100%
**7**	We recommend interrupting contact between the exposed child and the patient with tuberculosis for at least 2 weeks or until the adult no longer harbors the bacterium.	9.35	1.18	10	9	10	5	10	IIIb	D	98%
**8**	If it is considered that there is a risk exposure to tuberculosis and the TST and IGRA are negative, chemoprophylaxis for LTBI will be administered for 2 months (8–10 weeks).	8.75	1.41	9.5	8	10	1	10	IIIb	D	91%
**9**	In the event of a positive TST or IGRA result, a simple posterior-anterior and lateral chest X-ray should be performed (if not previously done).	9.76	0.63	10	10	10	7	10	IIIb	D	100%
**10**	If the IGRA and/or TST results are positive and tuberculosis is ruled out, latent tuberculous infection should be suspected, and appropriate treatment administered.	9.13	3.54	10	9	10	5	10	IIIb	D	93%
**11**	Latent tuberculosis infection should be treated using any of the following regimens: • Isoniazid (H) for 6–9 months (6 H or 9 H). • Isoniazid and rifampicin (R) for 3 months (3 HR) or in children older than 12 years, isoniazid and rifapentine once weekly for 3 months (3HP) with directly observed therapy. • Rifampicin for 4 months (4 R).	9.00	1.60	10	9	10	5	10	IIIb	D	89%
**12**	An infectious disease specialist should be consulted for the management of patients with rheumatic disease and tuberculosis (exposure/infection/disease).	9.59	0.89	10	10	10	6	10	IIIb	D	98%
**13**	In non-endemic areas it is not necessary to take specific preventive measures against fungal infections before starting immunosuppressive treatment.	8.76	1.44	9	8	10	4	10	IIIb	D	91%
**14**	In the case of travel to areas that are endemic for *Histoplasma capsulatum*, *Blastomyces dermatitidis* and *Coccidioides immitis*, avoid risk activities (cave exploration, bird’s nest cleaning).	9.22	1.41	10	9	10	5	10	IIIb	D	91%
**15**	Primary antifungal prophylaxis with oral posaconazole could be considered in patients with severe neutropenia (< 500 cells/µL) for more than 1 week.	8.11	1.86	8	7	10	4	10	IIIb	D	80%
**16**	Consider prophylaxis against *Pneumocystis jirovecii* pneumonia in the following rheumatic disease: systemic vasculitis (granulomatosis with polyangiitis, polyarteritis nodosa), idiopathic inflammatory myopathies, and associated interstitial lung disease. It should also be considered in patients receiving sustained immunosuppressive treatment that combines high doses of corticosteroids and an immunosuppressant or 2 associated immunosuppressive drugs.	8.70	1.41	9	8	10	5	10	IIIb	D	87%
**17**	Consider prophylaxis against *Pneumocystis jirovecii* pneumonia in patients receiving long-term high-dose corticosteroids.	9.02	2.12	9	8	10	5	10	IIIb	D	96%
**18**	Prophylaxis for *Pneumocystis jirovecii* pneumonia is indicated in patients receiving cyclophosphamide to induce remission of ANCA-associated vasculitis.	9.11	3.54	10	9	10	5	10	IIIa	C	91%
**19**	*Pneumocystis jirovecii* prophylaxis in patients treated with rituximab should be considered in those receiving concomitant immunosuppressive therapy, including prednisone > 20 mg/day or equivalent dose for at least 4 weeks.	9.04	1.50	10	9	10	4	10	IIIa	C	91%
**20**	Prophylaxis for *Pneumocystis jirovecii* pneumonia in patients treated with TNF-α antagonists should only be considered in the presence of 2 or more of the following risk factors: high-dose corticosteroids, concomitant lung disease, persistent lymphopenia, hypoalbuminemia, and hypogammaglobulinemia.	8.63	1.73	9.5	8	10	5	10	IIIa	D	85%
**21**	The total lymphocyte counts below 500 cells/mm^3^ will be taken into consideration before initiation of prophylaxis for *Pneumocystis jirovecii* pneumonia.	7.78	2.22	8	5	10	3	10	IIIb	D	70%
**22**	The drug of choice for prevention of *Pneumocystis jiroveccii* pneumonia is trimethoprim-sulfamethoxazole at 5 mg/kg/day of trimethoprim, 3 days a week (on consecutive or alternative days).	9.69	0.62	10	10	10	7	10	IIIb	B	100%
**23**	For patients unable to tolerate trimethoprim-sulfamethoxazole, other prophylactic strategies include dapsone, atovaquone, and aerosolized pentamidine.	8.67	1.64	7	10	10	7	10	IIIb	D	100%

*MMR* measles, mumps, and rubella, *SD* standard deviation, *Min* minimum, *Max* maximum, *LE* level of evidence, *GR* grade of recommendation, *GA* grade of agreement, *IGRA* interferon-γ release assay, *TST* tuberculin skin test, *LTBI* latent tuberculosis infection, *ANCA* antineutrophil cytoplasmic antibody, *TNF-α* tumor necrosis factor α

*The level of evidence was based on the Oxford Center for Evidence-Based Medicine classification

#### Recommendation 1

Triple viral vaccination (measles, mumps, rubella (MMR)) is recommended, whenever possible, at least 2–4 weeks before starting immunosuppressive treatment (LE IIIb; GR D; LA 98%).

Children with rheumatic diseases, especially those receiving immunosuppressive treatment, are more susceptible to infections; therefore, it is important to update the vaccination schedule according to national vaccination guidelines. Because immunogenicity may be reduced by immunosuppressive treatments, usual practice and current consensus-based guidelines recommend waiting at least 2–4 weeks before starting these treatments after vaccination with live attenuated vaccines [2].

Booster vaccination against MMR is currently administered in patients receiving methotrexate < 15 mg/m^2^/week or low-dose corticosteroids. However, live attenuated vaccine is not recommended during disease flares or in patients taking high-dose corticosteroids (≥ 2 mg/kg or ≥ 20 mg/day for 2 weeks) or high doses of csDMARDs or bDMARDs. Nevertheless, recent data showed that booster MMR vaccine in patients with juvenile idiopathic arthritis receiving bDMARDs (anti-TNFα, anti-IL-1, or anti-IL-6) is safe and immunogenic, with no patients developing a disease flare [6, 7]. Therefore, vaccination can be considered on a case-to-case basis, weighing the risk of infections against the risk of inducing infection [8].

#### Recommendation 2

If serology testing for varicella zoster virus is negative, varicella vaccination is recommended, whenever possible, 2–4 weeks before starting immunosuppressive treatment (LE IIIb; GR D; LA 100%).

It is recommended to record varicella zoster virus (VZV) infection and vaccination history in children with rheumatic diseases, especially in those patients anticipating high-dose csDMARDS or bDMARDs. In cases of a negative history of infection or vaccination and a negative serology result during screening, VZV vaccine should be administered, at least 2–4 weeks before initiation of immunosuppressive therapy [2].

As VZV vaccine is a live attenuated vaccine, like MMR, the recommendations are similar to those described in the previous section. Increasing evidence from small case series shows that not only booster, but also primary VZV vaccination can be safe in children receiving treatments such as methotrexate, corticosteroids, and even bDMARDS (anti-TNF, anti-IL6) [9, 10].

#### Recommendation 3

Children 6 months or older with rheumatic diseases should be vaccinated against influenza during the flu season, especially if they are receiving immunosuppressive treatment. Vaccination of the entire family is recommended (LE IIIb; GR D; LA 98%).

Influenza vaccination combined with non-pharmacological measures continues to be the fundamental approach for preventing influenza today. The Vaccine Advisory Committee of the Spanish Association of Pediatrics recommends annual influenza vaccination in all chronically ill or immunosuppressed patients and their cohabiting family members provided they are over 6 months of age. Two doses separated by 4 weeks are administered in children under 9 years old who are being vaccinated for the first time the first year, and 1 dose if they are older than this age. In the following years, only 1 dose is needed each season.

The preparations currently marketed and approved for children are inactivated vaccines for intramuscular or subcutaneous administration (0.5 ml). At present, the most widely used preparations are the inactivated trivalent vaccines. However, the trend is towards the use of tetravalent preparations to optimize the effectiveness of influenza vaccination in the most vulnerable population [11]. An attenuated vaccine is currently marketed for intranasal administration (0.2 ml), although is contraindicated in immunocompromised patients.

Influenza vaccination in people receiving immunosuppressants may be less effective depending on the treatment administered [12–15]. Although the quality of the evidence is low, prophylaxis with oseltamivir for 10 days may be considered in immunosuppressed children at high risk of the complications of influenza, especially if unvaccinated, after close contact with a person infected by influenza virus [16].

### Varicella zoster

#### Recommendation 4

Prophylactic treatment for chickenpox is recommended after contact with a person with varicella zoster infection in children with rheumatic diseases receiving medium- and high-risk immunosuppressive treatments who do not have a previous history of chickenpox or herpes zoster, vaccination, or evidence of immunity confirmed by serology testing (LE IIIb; GR D; LA 91%).

Regarding VZV infection, the risk of immunosuppressive drugs can be classified as low, intermediate (individuals who should be able to develop and maintain adequate antibodies from a previous infection or vaccination), and high (if they have not been able to develop or maintain immunity, since these may have been lost through immunosuppressive treatment) (Table 2) [17].

**Table 2:** Risk of opportunistic infection with VZV due to immunosuppressive drugs

**Low risk**	**Medium risk**^**b**^	**High risk**^**c**^
**Prednisone**^**a**^	Prednisolone/Methylprednisolone: ≥ 40 mg/day for at least a week > 2 mg/kg/day for at least a week Prednisone: ≥ 2 mg/kg/day (20 mg in children ≥ 10 kg) for at least 2 weeks ≥ 20 mg/day for at least 2 weeks ≥ 1 mg/kg/day at least a month	bDMARDs
**Methotrexate**^**a**^	Methotrexate 15 mg/m^2^/week or > 0.4 mg/kg/week	JAK inhibitors
**Azathioprine**^**a**^	Azathioprine ≥ 3 mg/kg/day	
**Sulfasalazine**	Mercaptopurine ≥ 1.5 mg/kg/day	Cyclophosphamide
**Hydroxychloroquine**	Leflunomide	Cyclosporine

*bDMARDs*, biological disease-modifying antirheumatic drugs

^a^At lower doses than in the intermediate-risk group

^b^Any of the drugs in the previous 3 months

^c^Any of the drugs in the previous 6 months

**Fig. 1:**
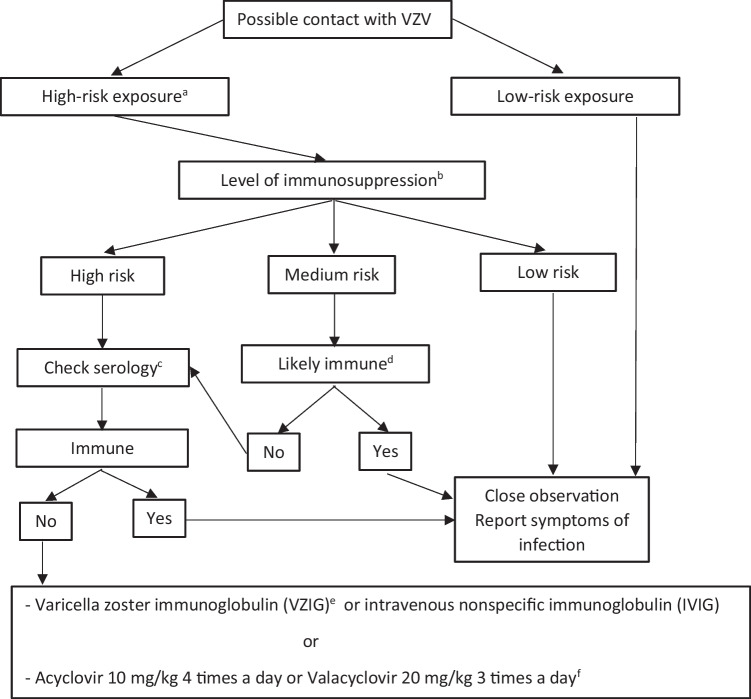
Algorithm for managing an immunosuppressed patient after contact with varicella zoster virus. ^a^Face to face or > 15 min in same room with any patient with chickenpox or exposed lesions (e.g., herpes zoster ophthalmicus) or contact with an immunosuppressed patient with covered zoster; ^b^See Table 2; ^c^If checking serology, do not delay treatment beyond 7 days post contact; It should be noted that serology might be unreliable ^d^History of chickenpox or shingles or varicella/shingles vaccination or prior serological evidence of immunity; ^e^VZV immunoglobulin is not frequently available (adapted from Cates M, Rheumatology 2018).^f^ If VZIG and IVIG are unavailable, contraindicated or a patient prefers not to receive a blood-derived product, acyclovir or vancyclovir could be used

An algorithm for the management of reported contact with VZV in an immunosuppressed patient is presented in Fig. 1. In low-risk patients, no action should be taken after contact with a patient with chickenpox, because the risk of serious disease caused by the medication is assumed to be low.

In intermediate-risk patients with a history of varicella or herpes zoster, prior VZV vaccination, or evidence of immunity by prior serology, no action should be taken. If this is not the case, serology testing should be performed. If this is negative, start treatment for exposure to chickenpox. Serology results should be available as early as possible, preferably before 7 days after exposure.

In all patients in the high-risk group, serology testing should be performed regardless of prior immunization status, vaccination, or positive serology after contact with chickenpox. This is because patients receiving immunosuppressive treatments in this group can reduce VZV-specific antibody titers to non-protective levels. This recommendation is based on expert opinion since the impact of specific immunosuppressants on VZV-specific humoral and cellular immunity is unknown [18, 19].

#### Recommendation 5

Prophylaxis with varicella zoster immunoglobulin or intravenous nons-pecific immunoglobulin within 10 days (preferably within 7 days) after exposure is recommended in patients considered at high risk of severe opportunistic disease (LE IIIb; GR D; LA 93%).

Administration of VZV immunoglobulin (VZIG) or intravenous non-specific immunoglobulin (IVIG) is recommended as soon as possible within 10 days (preferably within 7 days) after exposure to VZV in patients considered to be at high risk of severe disease (Fig. 1). VZIG is administered intramuscularly at 125 units/10 kg (maximum of 625 units) or according to the following dosage: 0–5 years, 250 mg; 6–10 years, 500 mg; 11–14 years, 750 mg; > 15 years, 1000 mg [17]. However, there are countries where this option is not available. Non-specific immunoglobulin is administered intravenously at 200 mg/kg.

For those seronegative contacts for whom VZIG is not indicated and/or those for whom prophylaxis with a non-blood product is preferred, oral acyclovir at 10 mg/kg 4 times a day (maximum dose of 3200 mg) or valacyclovir at 20 mg/kg 3 times a day (maximum dose 3000 mg) from days 7 to 14 after exposure can be considered. It should be noted that there is a risk of nephrotoxicity if cyclosporine is co-administered with acyclovir or valacyclovir. Acyclovir is excreted by the same tubular system as mycophenolate, although its co-administration does not produce a clinically significant change in levels in patients with normal renal function [20]. One small study has suggested an added benefit of acyclovir with VZIG compared with VZIG alone in children with renal disease taking steroids, however, in severely immunocompromised patients could be considered [19].

### Tuberculosis

#### Recommendation 6

In the event of close contact with a person with active tuberculosis, the tuberculin skin test (TST) and interferon-gamma release assay (IGRA) should be performed, as should a chest X-ray (LE IIIb; GR D; LA 100%).

A risk contact for tuberculosis is considered one that has occurred during the previous 3 months, with close contact (> 4 h daily in the same room) with confirmed smear-positive tuberculosis (pulmonary, laryngeal, tracheal, or endobronchial). In these cases, a TST and IGRA should be performed to detect latent tuberculosis infection (LTBI) or tuberculosis [21].

Both the TST and the IGRA depend on cell-mediated immunity and provide immunologic evidence of host sensitization to antigens of *Mycobacterium tuberculosis*. Neither method can distinguish between latent tuberculosis infection and tuberculosis, and both methods display suboptimal performance in immunocompromised patients, who are at greatest risk for progression of LTBI to TB [22]. Long-term treatment with corticosteroids (more than 7.5 mg prednisone equivalent) and other immunosuppressive drugs can significantly affect the performance of the IGRA, including sensitivity and indeterminate results, probably due to corticosteroid-induced lymphopenia or the impaired function of T cells and antigen-presenting cells [23].

Performing both the TST and the IGRA maximizes the sensitivity of the results in children of any age with suspected tuberculosis, although it is mandatory in children under biological therapy (especially TNF-α antagonists) when the initial and repeat IGRA results are indeterminate or invalid, when the initial test results (TST or IGRA) are negative but there is a high clinical suspicion of TB, and when an initial TST result is positive in children previously vaccinated against BCG [22, 24].

It is necessary to rule out TB by asking about compatible clinical signs and symptoms and, if present, by performing a chest X-ray (anteroposterior and lateral) [21]. Importantly, tuberculosis is often asymptomatic, and the physical findings of childhood tuberculosis are usually few and non-specific in mild and moderate forms of the disease. Computed tomography (CT) is more sensitive than plain radiographs, but its routine use is not recommended. Nevertheless, it is useful in children with inconclusive radiographic findings, symptomatic patients with normal radiographic features, and if the diagnosis is uncertain in at-risk groups [25].

#### Recommendation 7

We recommend interrupting contact between the exposed child and the patient with tuberculosis for at least 2 weeks or until the adult no longer harbours the bacterium (LE IIIb; GR D; LA 98%).

It is recommended that the patient with tuberculosis be kept in isolation in an independent room at home while starting treatment. Isolation should be maintained at least until it is verified that the adult with tuberculosis is not harboring the bacterium (usually estimated after at least 2 weeks of adequate treatment) [21].

#### Recommendation 8

If it is considered that there is a risk of exposure to tuberculosis and the TST and IGRA are negative, chemoprophylaxis for LTBI will be administered for 2 months (8–10 weeks) (LE IIIb; GR D; LA 91%).

Even with a negative TST and IGRA result after a risk contact for tuberculosis, especially in children with risk factors for false-negative/indeterminate TST/IGRA results, such as those younger than 5 years of age or those receiving immunosuppressive therapy, treatment should be started for possible LTBI (window or primary chemoprophylaxis). Primary chemoprophylaxis is usually administered with daily isoniazid (H) (Table 3) [21].

**Table 3:** Treatment regimens for latent tuberculosis infection

**Regimen**	**Agent(s)**	**Dose and administration**	**Duration (month)**
**6H**	Isoniazid	10–15 mg/kg daily (max 300 mg) SAT 20–30 mg/kg (max 900 mg) twice weekly DOT	6
**9H**	Isoniazid	10–15 mg/kg daily (max 300 mg) SAT 20–30 mg/kg (max 900 mg) twice weekly DOT	6
**3HR**	Isoniazid + Rifampicin	Same doses as when drugs are used individually	3
**3HP**	Isoniazid + Rifapentine	Isoniazid 15 mg/kg (age > 12 years) or 25 mg/kg (2–11 years) rounded up nearest 50 or 100 mg (max 900 mg) and Rifapentine 300 mg (10–14 kg), 450 mg (14.1–25 kg), 600 mg (25.1–32 kg), 750 mg (32.1–49.9) or 900 mg (> 50 kg) weekly SAT or DOT	3
**4R**	Rifampicin	15–20 mg/kg daily (max 600 mg) SAT	4

Table adapted from Nolt D, Pediatrics 2021

*SAT* self-administered therapy, *DOT* directly observed therapy

TST or IGRA should be repeated after 8 to 10 weeks. If the results are still negative, chemoprophylaxis can be discontinued. If the TST or IGRA result becomes positive, active disease will be ruled out again and the regimen for LTBI should be completed [22]. In immunocompromised children, full treatment of LTBI is recommended, owing to the possible absence of TST reactivity and indeterminate/invalid results of IGRA, even if repeated TST or IGRA at 8 weeks are negative [21].

#### Recommendation 9

In the event of a positive TST or IGRA result, a simple posterior-anterior and lateral chest X-ray should be performed (if not previously done) (LE IIIb; GR D; LA 100%).

TB must be ruled out in the event of a positive TST or IGRA result by a thorough physical examination and a posterior-anterior and lateral chest X-ray. In the presence of radiological or clinical findings, additional diagnostic procedures (induced sputum or gastric juice aspiration depending on age) should be performed to confirm pulmonary and/or extrapulmonary tuberculosis. When choosing the treatment regimen, the risk of developing drug-resistant tuberculosis while on monotherapy with isoniazid or rifampin should be considered [22].

#### Recommendation 10

If the IGRA and/or TST results are positive and tuberculosis is ruled out, latent tuberculosis infection (LTBI) should be suspected, and appropriate treatment administered (LE IIIb; GR D; LA 93%).

LTBI is suspected in asymptomatic patients with a normal chest X-ray and positive TST and/or IGRA findings and a known contact with a tuberculosis patient harboring the bacterium. However, in clinical practice, children with no known risk contact for tuberculosis but with a positive TST and/or IGRA result should also be considered as having LTBI, especially if they are under 5 years of age or immunosuppressed. In the absence of a known contact or risk factors and a history of BCG vaccination, a positive TST result with a negative IGRA result is interpreted as an effect of BCG. These cases are not considered LTBI [21].

All children and adolescents diagnosed with LTBI should receive treatment as soon as possible to prevent tuberculosis.

#### Recommendation 11

Latent tuberculosis infection should be treated with any of the following treatment regimens:

- Isoniazid (H) for 6–9 months (6H or 9H).
- Isoniazid and rifampicin (R) for 3 months (3HR) or in children older than 12 years, isoniazid and rifapentine once weekly for 3 months (3HP). Directly observed therapy may be offered in intermittent treatment for improving adherence.
- Rifampicin for 4 months (4R) (LE IIIb; GR D; LA 89%).

The treatment regimens for LTBI are described in Table 3. It should be noted that these regimens have not been specifically tested in immunocompromised patients. Adherence to medication regimens for LTBI can be improved with directly observed therapy or administration of medications by a health care professional or trained third party who observes and reports that the patient takes each dose of medication.

Isoniazid monotherapy (6H or 9H) is the most widely recommended and used treatment for LTBI, with an efficacy of 98% against tuberculosis. However, the long duration of this therapy (usually up to 9 months in immunocompromised patients) results in poor adherence and low completion rates. Therefore, it is only recommended when a rifampicin-containing regimen cannot be used.

Treatment regimens that combine isoniazid and rifampicin (3HR) or rifapentine 3HP have comparable or better efficacy than isoniazid in monotherapy, although they are associated with higher completion rates. 3HP is associated with good tolerance and low toxicity and is especially recommended in adolescents or when poor adherence is suspected.

The 4-month regimen of daily rifampicin (4R) has similar efficacy to 9 months of isoniazid, although the shorter period is associated with a significantly higher rate of completion. It is indicated in patients with toxicity or contraindications to isoniazid, or when strains of *Mycobacterium tuberculosis* resistant to isoniazid (and sensitive to rifampicin) are present [22, 26].

Due to the low risk of hepatotoxicity in children, routine transaminase monitoring is not recommended during LTBI treatment, except in the case of hepatotoxicity, underlying liver disease, or concomitant hepatotoxic medication. In immigrants from countries endemic for viral hepatitis or HIV, both diseases should be ruled out before starting treatment [21]. It is not necessary to repeat chest X-ray after completion. TST/IGRA remain positive and should not be repeated either [22].

The recommended duration of LTBI treatment before starting immunosuppressive therapy is not well established, although most authors suggest that, whenever possible, patients should receive at least 1 month of treatment for tuberculosis before starting immunosuppressive therapy.

#### Recommendation 12

An infectious disease specialist should be consulted for the management of patients with rheumatic disease and tuberculosis (exposure/infection/disease) (LE IIIb; GR D; LA 98%).

A specialist in infectious diseases should be consulted when test results are difficult to interpret. When TST or IGRA is positive, the specialist should collaborate in the early reporting of cases to the public health authorities [22]. An infection caused by a strain of *Mycobacterium tuberculosis* that is resistant to isoniazid and rifampicin (or to other antituberculosis drugs) requires an individual approach based on the exact drug resistance pattern. These cases should also be managed in consultation with a specialist with expertise in managing pediatric tuberculosis [27].

### Fungal infections

#### Recommendation 13

In non-endemic areas, it is not necessary to take specific preventive measures against fungi before starting immunosuppressive treatment (LE IIIb; GR D; LA 91%).

The risk of opportunistic and endemic invasive fungal diseases (IFD) appears to depend on the underlying condition, the concomitant administration of corticosteroids and immunosuppressants, and geographic exposure. The recommendation to perform serology testing for *Histoplasma capsulatum*, *Blastomyces dermatitidis*, and *Coccidioides immitis* prior to starting immunosuppressive treatment would only be indicated in patients from endemic areas (Sub-Saharan Africa, Latin America, Southeast Asia) [28, 29].

#### Recommendation 14

In the case of travel to areas that are endemic for *Histoplasma capsulatum*, *Blastomyces dermatitidis*, and *Coccidioides immitis*, avoid risk activities (cave exploration, bird’s nest cleaning) (LE IIIb; GR D; LA 91%).

To avoid possible IFD in children with immunosuppressive treatment, risk activities such as cave exploration or nest cleaning should be avoided if travelling to areas that are endemic for *Histoplasma capsulatum*, *Blastomyces dermatitidis*, and *Coccidioides immitis* (Sub-Saharan Africa, Latin America, Southeast Asia) [28, 29].

#### Recommendation 15

Primary antifungal prophylaxis with oral posaconazole could be considered in patients with severe neutropenia (< 500 cells/µL) for more than 1 week (LE IIIb; GR D; LA 80%).

In patients with hematologic malignancies, primary antifungal prophylaxis with oral posaconazole is used in patients with severe neutropenia (< 500 cells/µL) lasting more than 1 week. The dose used is 200 mg every 8 h in children over 13 years of age. The alternative to posaconazole would be voriconazole administered orally (9 mg/kg/12 h in < 50 kg, 4 mg/kg/12 h in > 50 kg) or intravenously (8 mg/kg/12 h in < 50 kg, 4 mg/kg/12 h in > 50 kg). In the case of contraindication or adverse effects with azoles, liposomal amphotericin B or intravenous micafungin could be considered [30].

Although IFD is very rare in rheumatic diseases, isolated cases have been described in adults with rheumatoid arthritis and systemic lupus erythematosus (SLE) [31–33]. In patients with prolonged neutropenia that may be caused by immunosuppressive treatment or by high disease activity, primary fungal prophylaxis could also be considered. However, this is one of the recommendations that achieved the lowest degree of agreement among the experts. In cases of suspected or documented IFD, a high index of clinical suspicion and prompt initiation of appropriate antifungal therapy are probably better tools for controlling morbidity and mortality from IFD in immunocompromised children.

Neutropenia secondary to tocilizumab has been reported, although it has not been associated with the development of bacterial or fungal infections [34].

### Pneumocystis pneumonia

#### Recommendation 16

Consider prophylaxis against *Pneumocystis jirovecii* pneumonia (PJP) in the following cases: systemic vasculitis (granulomatosis with polyangiitis, polyarteritis nodosa), idiopathic inflammatory myopathies, and associated interstitial lung disease. It should also be considered in patients receiving sustained immunosuppressive treatment that combines high doses of corticosteroids and an immunosuppressant or 2 associated immunosuppressive drugs (LE IIIb; GR D; LA 87%).

*Pneumocystis jirovecii* is a ubiquitous fungus that can cause pneumonia (PJP) in immunocompromised individuals, such as children with human immunodeficiency virus (HIV) infection, children with cancer, organ transplant recipients, and children with underlying autoimmune rheumatic diseases [35]. The overall incidence of PCP in patients with autoimmune rheumatic diseases remains relatively low, and PCP is usually the result of the simultaneous combination of several risk factors, such as immunosuppressive treatment, lymphopenia, and interstitial lung disease. However, when PCP occurs, morbidity and mortality are usually high [36–38].

The rheumatic disease with the highest risk for PJP is granulomatosis with polyangiitis (GPA), although it has also been reported in polyarteritis nodosa, idiopathic inflammatory myopathies (dermatomyositis, polymyositis), and autoimmune disease with associated interstitial lung disease. PJP prophylaxis should also be considered in any patient with an autoimmune disease in whom treatment is expected to be long-term. This should take the form of high doses of prednisone combined with an immunosuppressive agent (mainly cyclophosphamide, methotrexate, TNF-α antagonists, and rituximab) or 2 immunosuppressive agents combined [39, 40].

In these cases, PJP prophylaxis has been reported to be effective and safe [37, 41]. A recent study has shown the variability of PJP prophylaxis in patients who have risk conditions or take high-risk immunosuppressants, with up to 25% not receiving prophylaxis. However, the rates of PJP reported are low (2.2%), and the probability of adverse effects of prophylaxis necessitates a more personalized risk assessment before prescribing PJP prophylaxis [42].

#### Recommendation 17

Consider prophylaxis against *Pneumocystis jirovecii* pneumonia (PJP) in patients receiving long-term high-dose corticosteroids (LE IIIb; GR D; LA 96%).

The main risk factor for the development of PJP is treatment with high-dose corticosteroids over long periods, such as prednisone > 0.4 mg/kg/day (or > 30 mg/day) for more than 4 weeks or > 0.2 mg/kg (or 15 mg/day) for more than 8 weeks [39, 43]. However, in a retrospective cohort that included 119,399 children who had received at least 2 prescriptions for a systemic corticosteroid within a 60-day period (cancer, transplant, and HIV were excluded), it was observed that the incidence of PJP was low (0.61 and 0.53/10,000 patient-years between children exposed and not exposed to PJP prophylaxis, respectively). Although favorable in adults with rheumatic diseases [37], the risk–benefit ratio of TMP/SMX prophylaxis in children treated with corticosteroids has been debated, given the possible incidence of adverse effects (e.g., skin reactions and myelosuppression) reported with this compound and the low incidence of PJP [36, 44].

PJP prophylaxis should be discontinued when prednisone doses reach < 15 mg/day, provided that no other associated risk factors remain (immunosuppressive agents combined, low total and CD4 + lymphocyte counts) [39, 43].

#### Recommendation 18

Prophylaxis for PJP is recommended in patients receiving cyclophosphamide to induce remission of ANCA-associated vasculitis (LE IIIa; GR C; LA 91%).

Although it can appear years after initiation of treatment, PJP has been more frequently reported in patients with GPA during therapy to induce remission [45, 46]. However, it has not been routinely indicated in randomized trials but rather was optional or suggested, depending on the decision of the researcher. Some authors recommend prophylaxis when the CD4 count is below 250/mm^3^ or 300/mm^3^. In other studies, prophylaxis was prescribed to all participants [47, 48]. To standardize management, EULAR guidelines routinely encourage the prescription of PJP prophylaxis in the early phase of induction therapy with cyclophosphamide in ANCA-associated vasculitis [49].

#### Recommendation 19

*Pneumocystis jirovecii* prophylaxis in patients treated with rituximab might be considered in those receiving concomitant immunosuppressive therapy, including prednisone > 20 mg/day or equivalent dose for at least 4 weeks (LE IIIb; GR C; LA 91%).

Rituximab is associated with the development of PJP in patients with autoimmune diseases, such as rheumatoid arthritis [50], GPA [51], and systemic lupus erythematosus [52], although it is much less frequent than in patients with lymphoproliferative diseases. PCP usually occurs within 3–6 months of the last rituximab infusion, although it could also occur up to 32 months after the last treatment, both in monotherapy and in combination with other immunosuppressive drugs [39, 53].

The indication for prophylaxis in patients with autoimmune diseases treated with rituximab must be individualized and should be considered, especially if patients receive corticosteroids at > 20 mg/day [54] or if other risk factors for PJP are present (old age, kidney or lung involvement, previous infections due to T cell–mediated immune dysfunction, lymphocytopenia, and low CD4 cell counts) [51, 53]. PJP prophylaxis could be limited to 6 months after rituximab infusion if no other immunosuppressive drugs are prescribed [54].

#### Recommendation 20

Prophylaxis for *Pneumocystis jirovecii* pneumonia in patients treated with TNF-α antagonists might be considered in the presence of 2 or more of the following risk factors: high-dose corticosteroids, concomitant chronic lung disease, persistent lymphopenia, hypoalbuminemia, and hypogammaglobulinemia (LE IIIa; GR D; LA 85%).

Although PCP has been reported in adult patients treated with TNF-α antagonists, its incidence is usually low, ranging from < 0.01/1000 person-years in the USA to 8.8/1000 person-years in Japan, and routine PJP prophylaxis may not be beneficial [55]. In reported cases, in addition to older age, 2 or more of the following risk factors were present: high-dose corticosteroids, persistent lymphopenia, concomitant lung disease, hypoalbuminemia, and hypogammaglobulinemia [56–58]. Although the evidence is only available from studies in adult population, the presence of several of these risk factors could be considered to individualize the need for prophylaxis.

#### Recommendation 21

The total lymphocyte count (< 500 cells/mm^3^) will be taken into consideration before initiation of prophylaxis for *Pneumocystis jirovecii* pneumonia (LE IIIb; GR D; LA 70%).

Lymphopenia and a low CD4 + cell count have been consistently reported as risk factors for PCP, and several studies have addressed the recommendation of PJP prophylaxis in non-HIV-infected patients based on total lymphocyte and/or CD4 + lymphocyte count. The limits usually considered for PJP prophylaxis are a lymphocyte count of < 500/mm^3^ and CD4 cell count of < 200/mm^3^ [31, 59, 60], although PCP may also occur at higher counts. In fact, the value of lymphocyte and CD4 counts in establishing the need for prophylaxis in patients with autoimmune diseases has been questioned, and more than 600 lymphocytes/mm^3^ and more than 300 CD4/mm^3^ [61]. These limits may be more useful in other types of non-HIV-infected patients, such as those with lymphoproliferative diseases or transplant recipients, than in those with autoimmune disease.

Nevertheless, in a survey of rheumatologists’ practice for prescribing PJP prophylaxis, only 15% of respondents considered peripheral lymphocyte counts and 7.5% monitored CD4 cell counts [62].

#### Recommendation 22

The drug of choice for prophylaxis if *Pneumocystis jirovecii* pneumonia is trimethoprim-sulfamethoxazole at 5 mg/kg/day of trimethoprim, 3 days a week (on consecutive or alternate days) (LE IIb; GR B; LA 100%).

Trimethoprim-sulfamethoxazole is the preferred agent for PJP prophylaxis (Table 4). Although several dosing regimens are used, administration 3 times a week is the most common since it is as effective as daily administration [63, 64]. In addition to its activity against *Pneumocystis jirovecii*, trimethoprim-sulfamethoxazole also confers protection against many bacterial infections and toxoplasmosis. The most common adverse reaction is rash, which may be severe (e.g., Stevens-Johnson syndrome and toxic epidermal necrolysis). In these cases, TMP-SMX should be permanently discontinued [35].

**Table 4:** Prophylaxis for *Pneumocystis jirovecii* pneumonia in children

Drug	Dose and administration	Adverse events	Comments
Trimethoprim-sulfamethoxazole (TMP-SMX)	5 mg/kg/day or 150 mg/m^2^ of trimethoprim (max 160 mg), 3 times weekly (on consecutive or alternate days) VO or IV	Hypersensitivity reactions (rashes, fever), neutropenia, hyperkalemia, increased transaminases, renal failure	Preferred prophylaxis regimen
Pentamidine	4 mg/kg (max 300 mg) every 4 weeks IV 300 mg every 4 weeks INH	Nephrotoxicity, hyper or hypokalemia, hypoglycemia, hypocalcemia, arrhythmias, pancreatitis, respiratory symptoms	Children > 2 years (IV) or > 5 years (INH)
Dapsone	2 mg/kg (max 100 mg) daily or 4 mg/kg (max 200 mg) weekly VO	Rash, fever, lymphadenopathy, hemolytic anemia, increased transaminases, methemoglobinemia	Children > 1 month
Atovaquone	1–3 months: 30 mg/kg daily 4–24 months: 45 mg/kg (max 1500 mg) daily > 2 years: 30 mg/kg (max 1500 mg) daily VO	Nausea, diarrhea, fever, hepatitis, rash	Pediatric data is limited

*VO* orally, *IV* intravenously, *INH* inhaled

#### Recommendation 23

For patients unable to tolerate trimethoprim-sulfamethoxazole, other prophylactic strategies include dapsone, atovaquone, and aerosolized pentamidine (LE IIIb; GR D; LA 100%).

In the case of adverse effects or intolerance to trimethoprim-sulfamethoxazole, the drug of choice for prophylaxis would be atovaquone [65]. Other options include nebulized pentamidine, provided that it can be administered under biosafety conditions, and dapsone, depending on the adverse effect profile (Table 4) [35]. It is advisable to reconsider the need for prophylaxis before starting it.

## Discussion

Based on the best available evidence and consensus of experts, we present a series of recommendations concerning common and rare scenarios in the prophylaxis of infection for patients with immune-mediated rheumatic diseases receiving immunosuppressive treatment.

Our review showed that the level of evidence is very low for most scenarios. Therefore, several recommendations were based on expert opinion.

The recommendations are intended to assist specialists involved in the care of these patients in their routine clinical practice. The availability of explicit recommendations related to common situations with immunosuppressive treatments could prove helpful in daily practice.

We would like to mention here some specific recommendations that were not included in the Delphi.

- Pneumococcal vaccination with PCV10 or PCV13 is recommended in all non-vaccinated pediatric patients with autoimmune/inflammatory rheumatic diseases. The immunogenicity and safety of PCVs have also been shown in these patients. The question remains whether should additionally receive a booster vaccination with the PPSV-23 in addition to the PCV10/13 vaccination. The EULAR Task Force decided after an intense discussion, that a 5-yearly PPSV-23 is not recommended as standard of care but can be considered in immunosuppressed patients and SLE patients. The taskforce recommends avoiding the PPSV-23 in cryopyrin-associated periodic syndrome due to safety reasons [ 66].
- Meningococcal vaccination: Meningococcal C (MenC) conjugate vaccination was shown to be immunogenic and safe in patients with rheumatic diseases. The MenACWY conjugate vaccine was immunogenic in most inflammatory disease patients, but seroprotection was lower in patients using anti-TNF agents. Therefore, an extra booster MenACWY vaccination should be considered [ 67].
- SARS-CoV2 vaccination: no patients had a significant increase in disease activity after vaccination, and the commonest side effects (localized pain and fatigue) were mild with no serious adverse events, with similar frequency in patients and controls. Most patients become seropositive after two doses of the vaccine; however, antibody titres are significantly lower than healthy controls; hence, the recommendation that patients on immunosuppression receive an additional dose of vaccine as part of the primary course. Current studies provide support for the current clinical practice of offering the BNT162b2 vaccine to children or adolescents with rheumatic diseases without stopping their immunosuppression [ 68].
